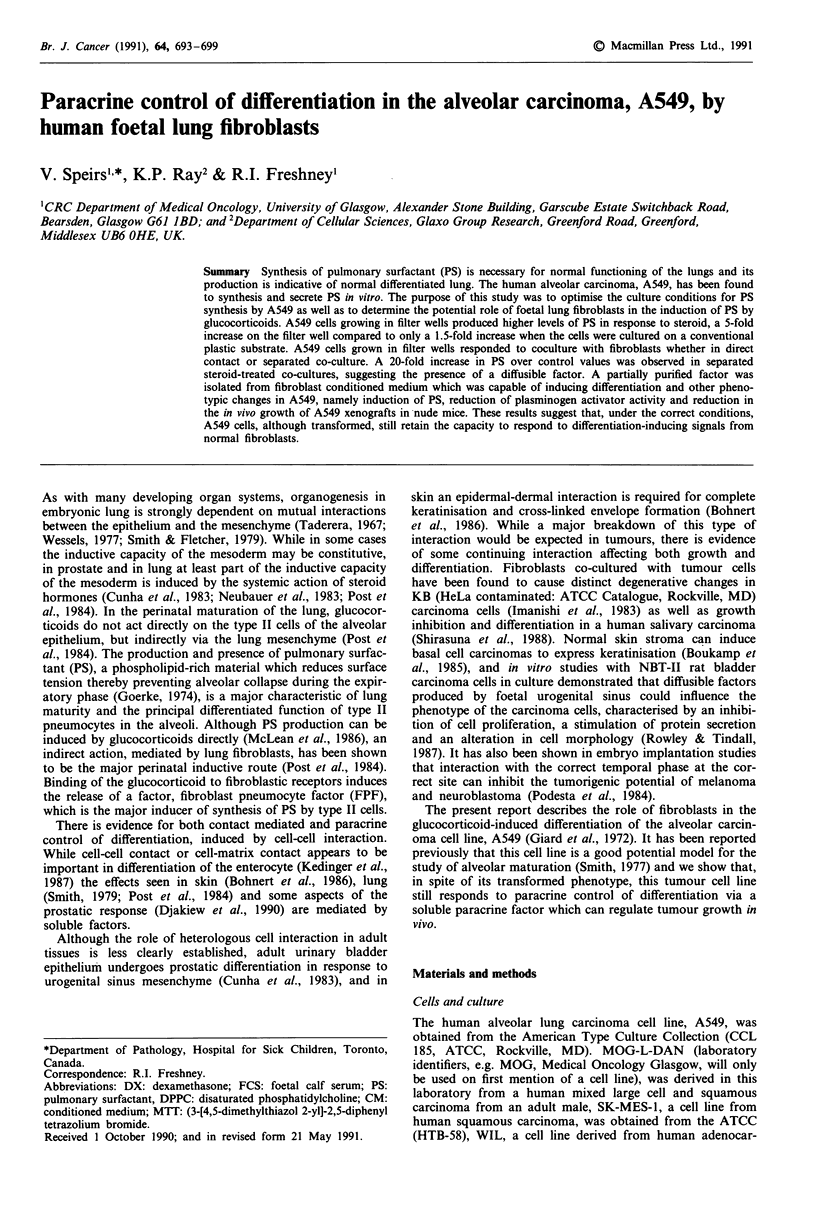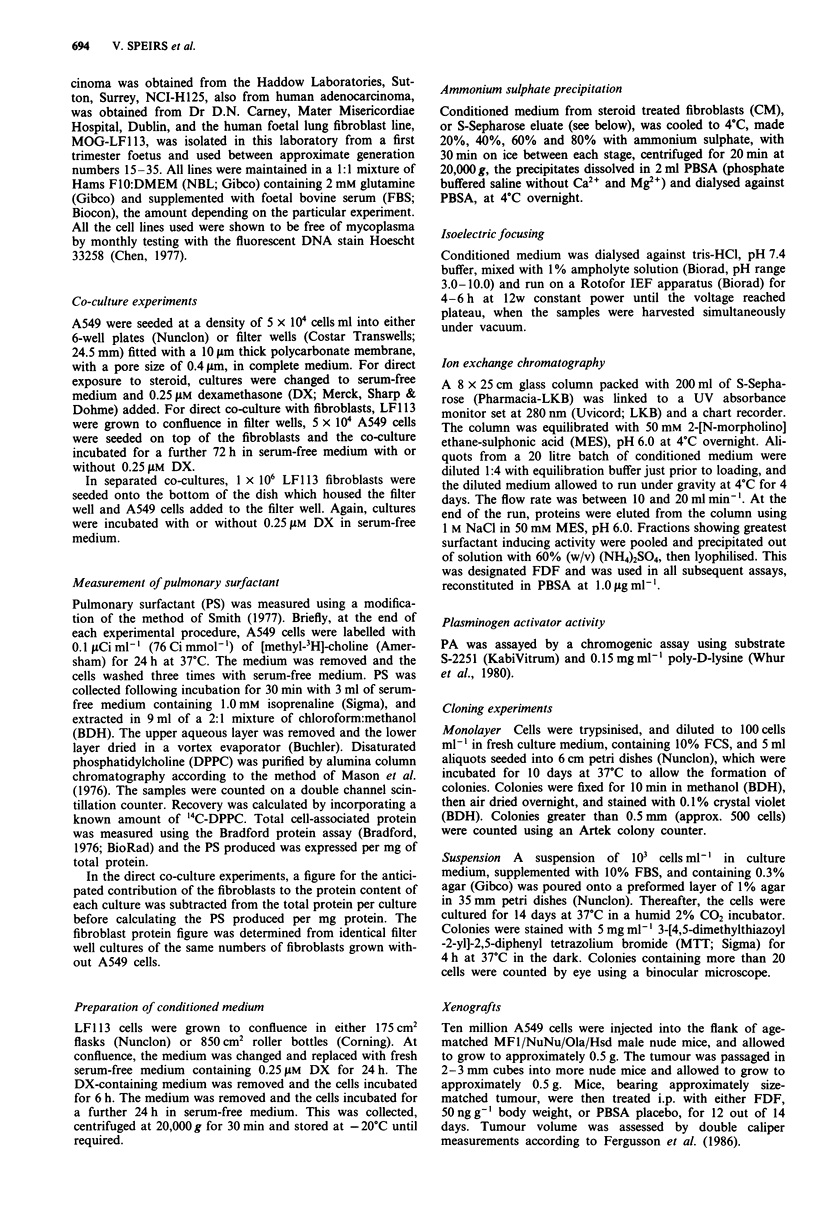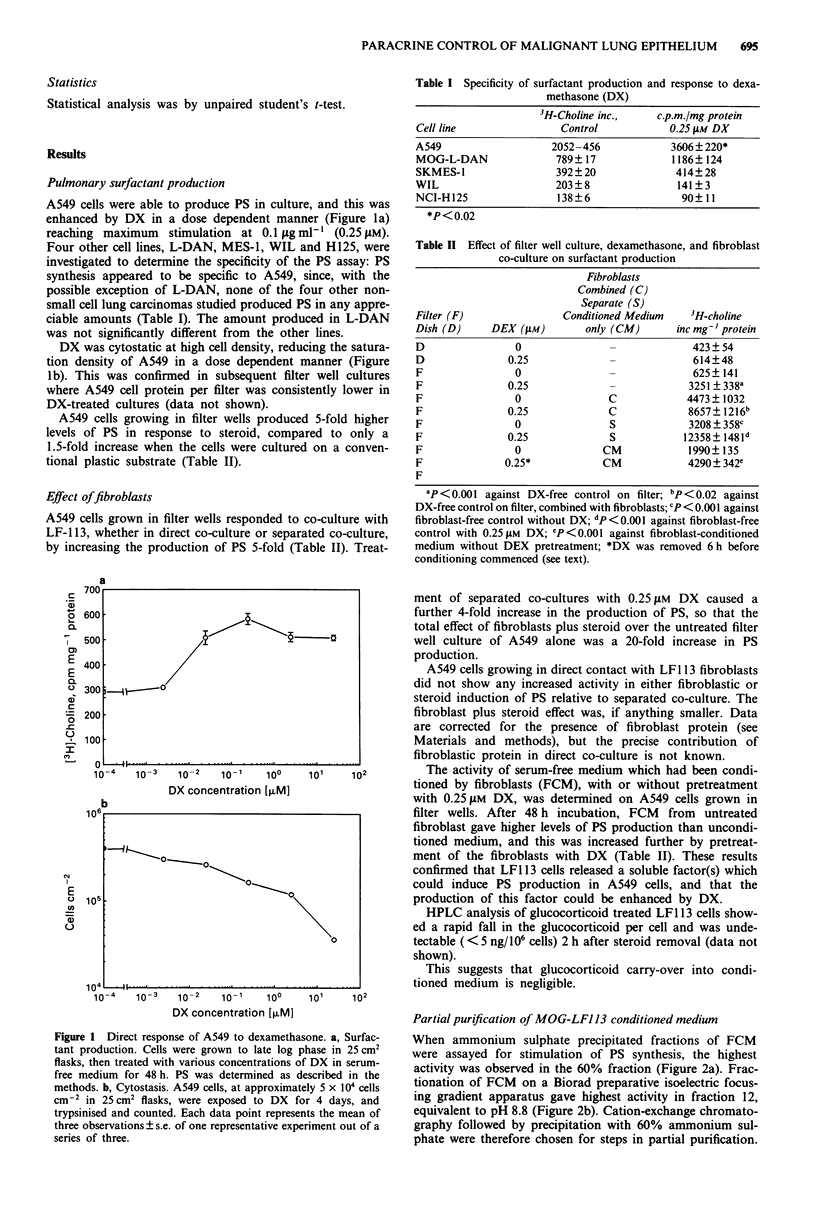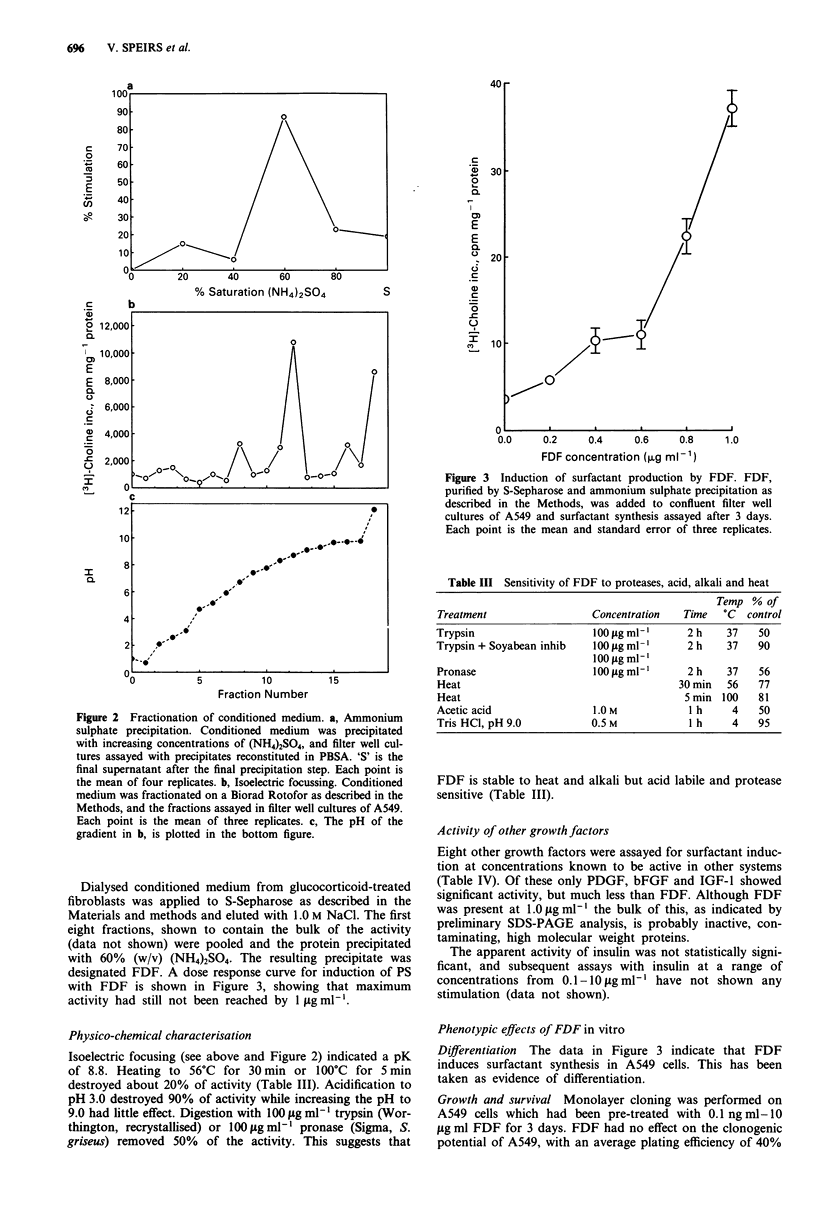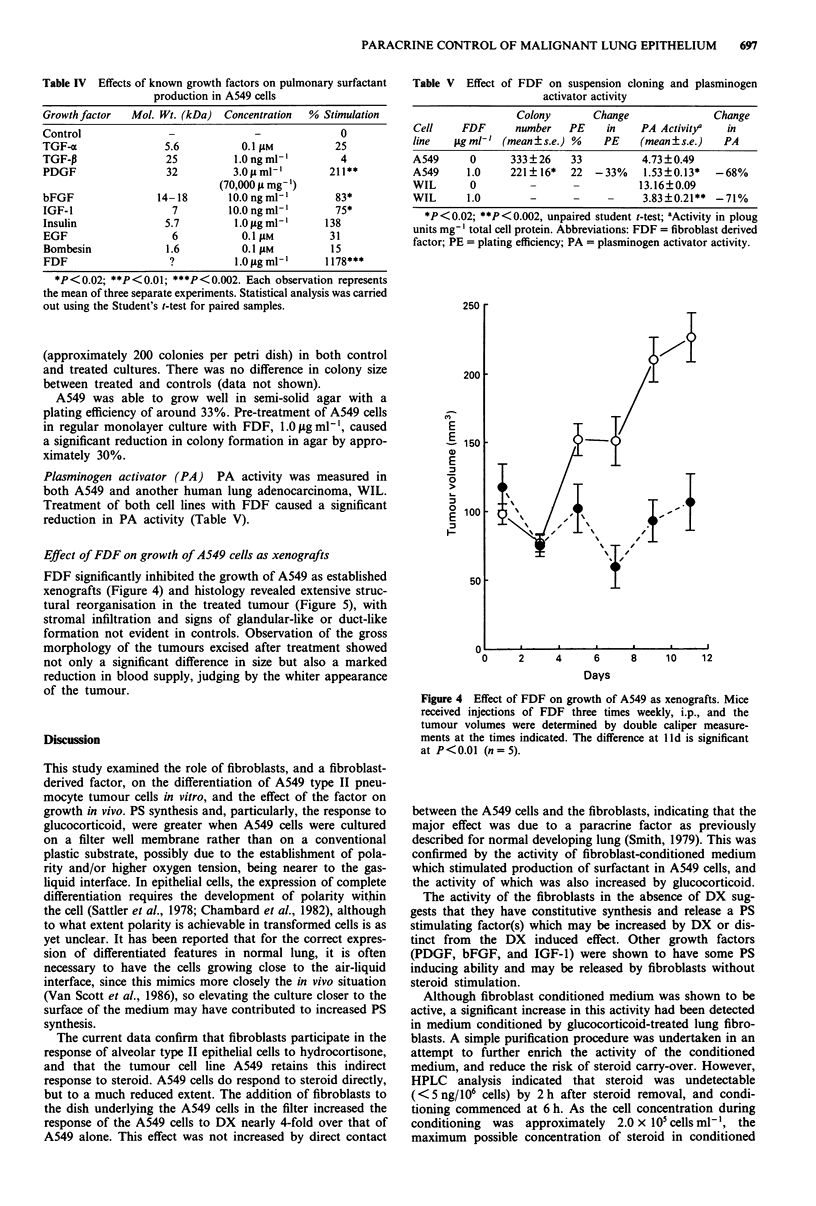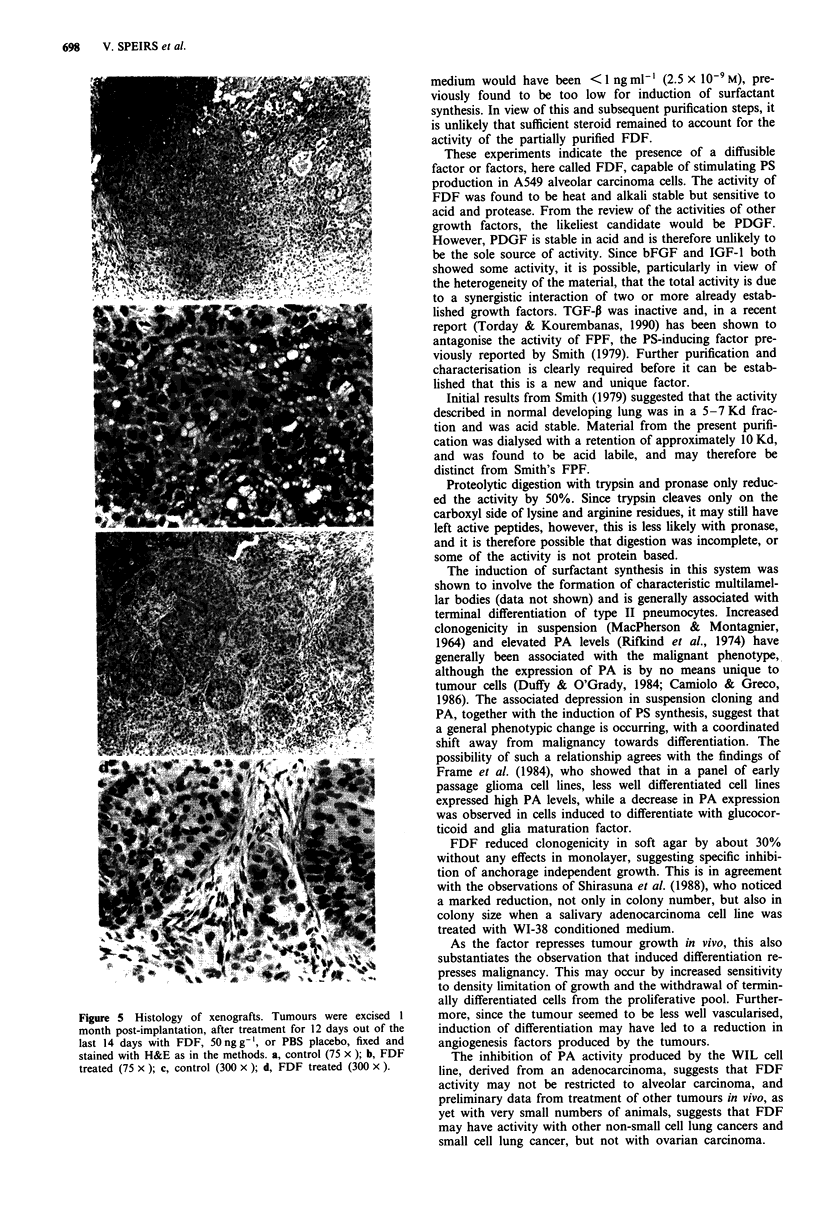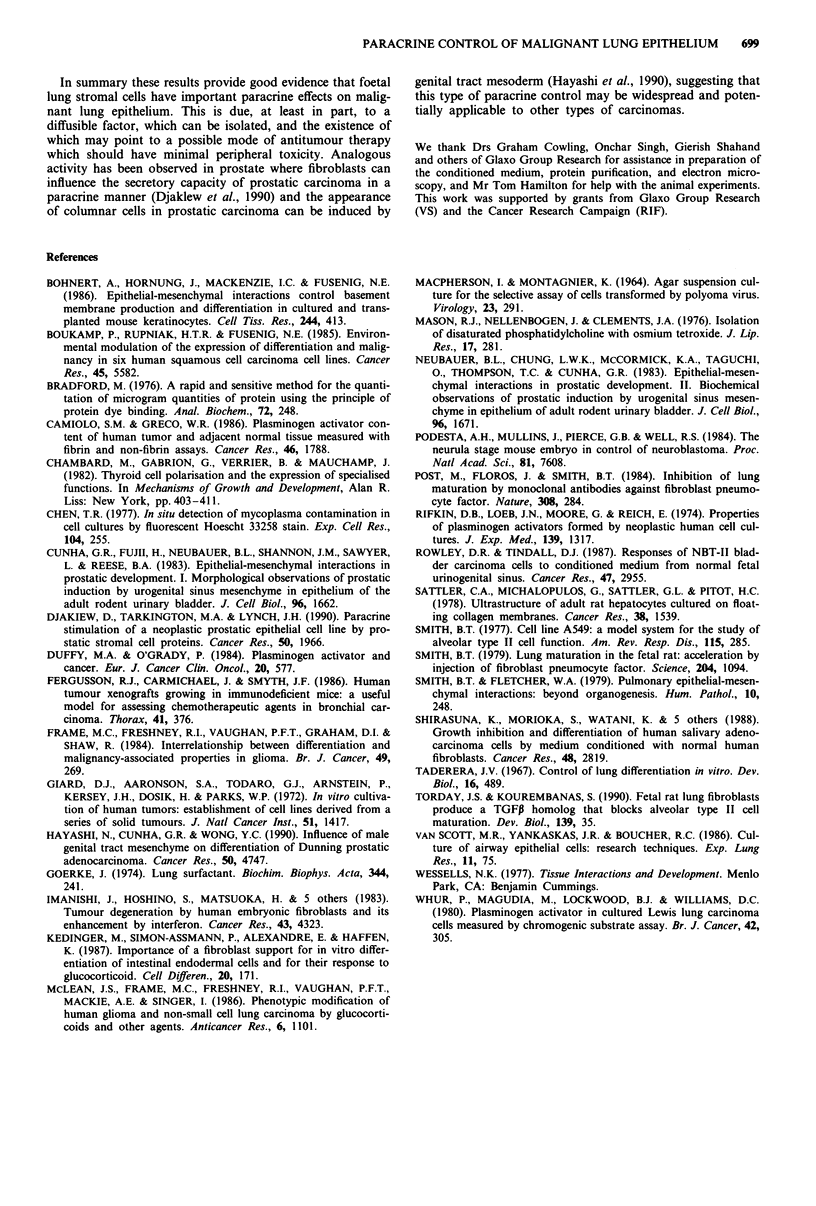# Paracrine control of differentiation in the alveolar carcinoma, A549, by human foetal lung fibroblasts.

**DOI:** 10.1038/bjc.1991.383

**Published:** 1991-10

**Authors:** V. Speirs, K. P. Ray, R. I. Freshney

**Affiliations:** CRC Department of Medical Oncology, University of Glasgow, Bearsden, UK.

## Abstract

**Images:**


Br. J. Cancer (1991), 64, 693-699 © Macmillan Press Ltd., 1991

### Paracrine control of differentiation in the alveolar carcinoma, A549, by human foetal lung fibroblasts

V. Speirs[1,*], K.P. Ray[2] & R.I. Freshney[1]

[1]*CRC Department of Medical Oncology, University of Glasgow, Alexander Stone Building, Garscube Estate Switchback Road, Bearsden, Glasgow G61 1BD; and* [2]*Department of Cellular Sciences, Glaxo Group Research, Greenford Road, Greenford, Middlesex UB6 0HE, UK.*

**Summary** Synthesis of pulmonary surfactant (PS) is necessary for normal functioning of the lungs and its production is indicative of normal differentiated lung. The human alveolar carcinoma, A549, has been found to synthesis and secrete PS *in vitro*. The purpose of this study was to optimise the culture conditions for PS synthesis by A549 as well as to determine the potential role of foetal lung fibroblasts in the induction of PS by glucocorticoids. A549 cells growing in filter wells produced higher levels of PS in response to steroid, a 5-fold increase on the filter well compared to only a 1.5-fold increase when the cells were cultured on a conventional plastic substrate. A549 cells grown in filter wells responded to coculture with fibroblasts whether in direct contact or separated co-culture. A 20-fold increase in PS over control values was observed in separated steroid-treated co-cultures, suggesting the presence of a diffusible factor. A partially purified factor was isolated from fibroblast conditioned medium which was capable of inducing differentiation and other phenotypic changes in A549, namely induction of PS, reduction of plasminogen activator activity and reduction in the *in vivo* growth of A549 xenografts in nude mice. These results suggest that, under the correct conditions, A549 cells, although transformed, still retain the capacity to respond to differentiation-inducing signals from normal fibroblasts.

As with many developing organ systems, organogenesis in embryonic lung is strongly dependent on mutual interactions between the epithelium and the mesenchyme (Taderera, 1967; Wessels, 1977; Smith & Fletcher, 1979). While in some cases the inductive capacity of the mesoderm may be constitutive, in prostate and in lung at least part of the inductive capacity of the mesoderm is induced by the systemic action of steroid hormones (Cunha *et al*., 1983; Neubauer *et al*., 1983; Post *et al*., 1984). In the perinatal maturation of the lung, glucocorticoids do not act directly on the type II cells of the alveolar epithelium, but indirectly via the lung mesenchyme (Post *et al*., 1984). The production and presence of pulmonary surfactant (PS), a phospholipid-rich material which reduces surface tension thereby preventing alveolar collapse during the expiratory phase (Goerke, 1974), is a major characteristic of lung maturity and the principal differentiated function of type II pneumocytes in the alveoli. Although PS production can be induced by glucocorticoids directly (McLean *et al*., 1986), an indirect action, mediated by lung fibroblasts, has been shown to be the major perinatal inductive route (Post *et al*., 1984). Binding of the glucocorticoid to fibroblastic receptors induces the release of a factor, fibroblast pneumocyte factor (FPF), which is the major inducer of synthesis of PS by type II cells.

There is evidence for both contact mediated and paracrine control of differentiation, induced by cell-cell interaction. While cell-cell contact or cell-matrix contact appears to be important in differentiation of the enterocyte (Kedinger *et al*., 1987) the effects seen in skin (Bohnert *et al*., 1986), lung (Smith, 1979; Post *et al*., 1984) and some aspects of the prostatic response (Djakiew *et al*., 1990) are mediated by soluble factors.

Although the role of heterologous cell interaction in adult tissues is less clearly established, adult urinary bladder epithelium undergoes prostatic differentiation in response to urogenital sinus mesenchyme (Cunha *et al*., 1983), and in skin an epidermal-dermal interaction is required for complete keratinisation and cross-linked envelope formation (Bohnert *et al*., 1986). While a major breakdown of this type of interaction would be expected in tumours, there is evidence of some continuing interaction affecting both growth and differentiation. Fibroblasts co-cultured with tumour cells have been found to cause distinct degenerative changes in KB (HeLa contaminated: ATCC Catalogue, Rockville, MD) carcinoma cells (Imanishi *et al*., 1983) as well as growth inhibition and differentiation in a human salivary carcinoma (Shirasuna *et al*., 1988). Normal skin stroma can induce basal cell carcinomas to express keratinisation (Boukamp *et al*., 1985), and *in vitro* studies with NBT-II rat bladder carcinoma cells in culture demonstrated that diffusible factors produced by foetal urogenital sinus could influence the phenotype of the carcinoma cells, characterised by an inhibition of cell proliferation, a stimulation of protein secretion and an alteration in cell morphology (Rowley & Tindall, 1987). It has also been shown in embryo implantation studies that interaction with the correct temporal phase at the correct site can inhibit the tumorigenic potential of melanoma and neuroblastoma (Podesta *et al*., 1984).

The present report describes the role of fibroblasts in the glucocorticoid-induced differentiation of the alveolar carcinoma cell line, A549 (Giard *et al*., 1972). It has been reported previously that this cell line is a good potential model for the study of alveolar maturation (Smith, 1977) and we show that, in spite of its transformed phenotype, this tumour cell line still responds to paracrine control of differentiation via a soluble paracrine factor which can regulate tumour growth *in vivo*.

### Materials and methods

### Cells and culture

The human alveolar lung carcinoma cell line, A549, was obtained from the American Type Culture Collection (CCL 185, ATCC, Rockville, MD). MOG-L-DAN (laboratory identifiers, e.g. MOG, Medical Oncology Glasgow, will only be used on first mention of a cell line), was derived in this laboratory from a human mixed large cell and squamous carcinoma from an adult male, SK-MES-1, a cell line from human squamous carcinoma, was obtained from the ATCC (HTB-58), WIL, a cell line derived from human adenocar-

*Department of Pathology, Hospital for Sick Children, Toronto, Canada.
Correspondence: R.I. Freshney.
Abbreviations: DX: dexamethasone; FCS: foetal calf serum; PS: pulmonary surfactant, DPPC: disaturated phosphatidylcholine; CM: conditioned medium; MTT: (3-[4,5-dimethylthiazol 2-yl]-2,5-diphenyl tetrazolium bromide.
Received 1 October 1990; and in revised form 21 May 1991.

cinoma was obtained from the Haddow Laboratories, Sutton, Surrey, NCI-H125, also from human adenocarcinoma, was obtained from Dr D.N. Carney, Mater Misericordiae Hospital, Dublin, and the human foetal lung fibroblast line, MOG-LF113, was isolated in this laboratory from a first trimester foetus and used between approximate generation numbers 15–35. All lines were maintained in a 1:1 mixture of Hams F10:DMEM (NBL; Gibco) containing 2 mM glutamine (Gibco) and supplemented with foetal bovine serum (FBS; Biocon), the amount depending on the particular experiment. All the cell lines used were shown to be free of mycoplasma by monthly testing with the fluorescent DNA stain Hoescht 33258 (Chen, 1977).

*Co-culture experiments*

A549 were seeded at a density of $5 \times 10^4$ cells ml into either 6-well plates (Nunclon) or filter wells (Costar Transwells; 24.5 mm) fitted with a 10 $\mu$m thick polycarbonate membrane, with a pore size of 0.4 $\mu$m, in complete medium. For direct exposure to steroid, cultures were changed to serum-free medium and 0.25 $\mu$M dexamethasone (DX; Merck, Sharp & Dohme) added. For direct co-culture with fibroblasts, LF113 were grown to confluence in filter wells, $5 \times 10^4$ A549 cells were seeded on top of the fibroblasts and the co-culture incubated for a further 72 h in serum-free medium with or without 0.25 $\mu$M DX.

In separated co-cultures, $1 \times 10^6$ LF113 fibroblasts were seeded onto the bottom of the dish which housed the filter well and A549 cells added to the filter well. Again, cultures were incubated with or without 0.25 $\mu$M DX in serum-free medium.

*Measurement of pulmonary surfactant*

Pulmonary surfactant (PS) was measured using a modification of the method of Smith (1977). Briefly, at the end of each experimental procedure, A549 cells were labelled with 0.1 $\mu$Ci ml$^{-1}$ (76 Ci mmol$^{-1}$) of [methyl-$^3$H]-choline (Amersham) for 24 h at 37°C. The medium was removed and the cells washed three times with serum-free medium. PS was collected following incubation for 30 min with 3 ml of serum-free medium containing 1.0 mM isoprenaline (Sigma), and extracted in 9 ml of a 2:1 mixture of chloroform:methanol (BDH). The upper aqueous layer was removed and the lower layer dried in a vortex evaporator (Buchler). Disaturated phosphatidylcholine (DPPC) was purified by alumina column chromatography according to the method of Mason *et al.* (1976). The samples were counted on a double channel scintillation counter. Recovery was calculated by incorporating a known amount of $^{14}$C-DPPC. Total cell-associated protein was measured using the Bradford protein assay (Bradford, 1976; BioRad) and the PS produced was expressed per mg of total protein.

In the direct co-culture experiments, a figure for the anticipated contribution of the fibroblasts to the protein content of each culture was subtracted from the total protein per culture before calculating the PS produced per mg protein. The fibroblast protein figure was determined from identical filter well cultures of the same numbers of fibroblasts grown without A549 cells.

*Preparation of conditioned medium*

LF113 cells were grown to confluence in either 175 cm$^2$ flasks (Nunclon) or 850 cm$^2$ roller bottles (Corning). At confluence, the medium was changed and replaced with fresh serum-free medium containing 0.25 $\mu$M DX for 24 h. The DX-containing medium was removed and the cells incubated for 6 h. The medium was removed and the cells incubated for a further 24 h in serum-free medium. This was collected, centrifuged at 20,000 *g* for 30 min and stored at −20°C until required.

*Ammonium sulphate precipitation*

Conditioned medium from steroid treated fibroblasts (CM), or S-Sepharose eluate (see below), was cooled to 4°C, made 20%, 40%, 60% and 80% with ammonium sulphate, with 30 min on ice between each stage, centrifuged for 20 min at 20,000 *g*, the precipitates dissolved in 2 ml PBSA (phosphate buffered saline without $Ca^{2+}$ and $Mg^{2+}$) and dialysed against PBSA, at 4°C overnight.

*Isoelectric focusing*

Conditioned medium was dialysed against tris-HCl, pH 7.4 buffer, mixed with 1% ampholyte solution (Biorad, pH range 3.0–10.0) and run on a Rotofor IEF apparatus (Biorad) for 4–6 h at 12w constant power until the voltage reached plateau, when the samples were harvested simultaneously under vacuum.

*Ion exchange chromatography*

A 8 × 25 cm glass column packed with 200 ml of S-Sepharose (Pharmacia-LKB) was linked to a UV absorbance monitor set at 280 nm (Uvicord; LKB) and a chart recorder. The column was equilibrated with 50 mM 2-[N-morpholino] ethane-sulphonic acid (MES), pH 6.0 at 4°C overnight. Aliquots from a 20 litre batch of conditioned medium were diluted 1:4 with equilibration buffer just prior to loading, and the diluted medium allowed to run under gravity at 4°C for 4 days. The flow rate was between 10 and 20 ml min$^{-1}$. At the end of the run, proteins were eluted from the column using 1 M NaCl in 50 mM MES, pH 6.0. Fractions showing greatest surfactant inducing activity were pooled and precipitated out of solution with 60% (w/v) $(NH_4)_2SO_4$, then lyophilised. This was designated FDF and was used in all subsequent assays, reconstituted in PBSA at 1.0 $\mu$g ml$^{-1}$.

*Plasminogen activator activity*

PA was assayed by a chromogenic assay using substrate S-2251 (KabiVitrum) and 0.15 mg ml$^{-1}$ poly-D-lysine (Whur *et al.*, 1980).

*Cloning experiments*

*Monolayer* Cells were trypsinised, and diluted to 100 cells ml$^{-1}$ in fresh culture medium, containing 10% FCS, and 5 ml aliquots seeded into 6 cm petri dishes (Nunclon), which were incubated for 10 days at 37°C to allow the formation of colonies. Colonies were fixed for 10 min in methanol (BDH), then air dried overnight, and stained with 0.1% crystal violet (BDH). Colonies greater than 0.5 mm (approx. 500 cells) were counted using an Artek colony counter.

*Suspension* A suspension of $10^3$ cells ml$^{-1}$ in culture medium, supplemented with 10% FBS, and containing 0.3% agar (Gibco) was poured onto a preformed layer of 1% agar in 35 mm petri dishes (Nunclon). Thereafter, the cells were cultured for 14 days at 37°C in a humid 2% $CO_2$ incubator. Colonies were stained with 5 mg ml$^{-1}$ 3-[4,5-dimethylthiazoyl -2-yl]-2,5-diphenyl tetrazolium bromide (MTT; Sigma) for 4 h at 37°C in the dark. Colonies containing more than 20 cells were counted by eye using a binocular microscope.

*Xenografts*

Ten million A549 cells were injected into the flank of age-matched MF1/NuNu/Ola/Hsd male nude mice, and allowed to grow to approximately 0.5 g. The tumour was passaged in 2–3 mm cubes into more nude mice and allowed to grow to approximately 0.5 g. Mice, bearing approximately size-matched tumour, were then treated i.p. with either FDF, 50 ng g$^{-1}$ body weight, or PBSA placebo, for 12 out of 14 days. Tumour volume was assessed by double caliper measurements according to Fergusson *et al.* (1986).

### *Statistics*

Statistical analysis was by unpaired student's *t*-test.

### Results

### *Pulmonary surfactant production*

A549 cells were able to produce PS in culture, and this was enhanced by DX in a dose dependent manner (Figure 1a) reaching maximum stimulation at 0.1 $\mu g\ ml^{-1}$ (0.25 $\mu M$). Four other cell lines, L-DAN, MES-1, WIL and H125, were investigated to determine the specificity of the PS assay: PS synthesis appeared to be specific to A549, since, with the possible exception of L-DAN, none of the four other non-small cell lung carcinomas studied produced PS in any appreciable amounts (Table I). The amount produced in L-DAN was not significantly different from the other lines.

DX was cytostatic at high cell density, reducing the saturation density of A549 in a dose dependent manner (Figure 1b). This was confirmed in subsequent filter well cultures where A549 cell protein per filter was consistently lower in DX-treated cultures (data not shown).

A549 cells growing in filter wells produced 5-fold higher levels of PS in response to steroid, compared to only a 1.5-fold increase when the cells were cultured on a conventional plastic substrate (Table II).

### *Effect of fibroblasts*

A549 cells grown in filter wells responded to co-culture with LF-113, whether in direct co-culture or separated co-culture, by increasing the production of PS 5-fold (Table II). Treatment of separated co-cultures with 0.25 $\mu M$ DX caused a further 4-fold increase in the production of PS, so that the total effect of fibroblasts plus steroid over the untreated filter well culture of A549 alone was a 20-fold increase in PS production.

**Figure 1** Direct response of A549 to dexamethasone. **a**, Surfactant production. Cells were grown to late log phase in 25 $cm^2$ flasks, then treated with various concentrations of DX in serum-free medium for 48 h. PS was determined as described in the methods. **b**, Cytostasis. A549 cells, at approximately $5 \times 10^4$ cells $cm^{-2}$ in 25 $cm^2$ flasks, were exposed to DX for 4 days, and trypsinised and counted. Each data point represents the mean of three observations ± s.e. of one representative experiment out of a series of three.

**Table I** Specificity of surfactant production and response to dexamethasone (DX)

| *Cell line* | *³H-Choline inc., Control* | *c.p.m./mg protein 0.25 μM DX* |
|---|---|---|
| A549 | 2052–456 | 3606±220* |
| MOG-L-DAN | 789±17 | 1186±124 |
| SKMES-1 | 392±20 | 414±28 |
| WIL | 203±8 | 141±3 |
| NCI-H125 | 138±6 | 90±11 |

*$P<0.02$

**Table II** Effect of filter well culture, dexamethasone, and fibroblast co-culture on surfactant production

| *Filter (F) Dish (D)* | *DEX (μM)* | *Fibroblasts Combined (C) Separate (S) Conditioned Medium only (CM)* | *³H-choline inc mg⁻¹ protein* |
|---|---|---|---|
| D | 0 | – | 423±54 |
| D | 0.25 | – | 614±48 |
| F | 0 | – | 625±141 |
| F | 0.25 | – | 3251±338[a] |
| F | 0 | C | 4473±1032 |
| F | 0.25 | C | 8657±1216[b] |
| F | 0 | S | 3208±358[c] |
| F | 0.25 | S | 12358±1481[d] |
| F | 0 | CM | 1990±135 |
| F | 0.25* | CM | 4290±342[e] |
| F | | | |

[a]$P<0.001$ against DX-free control on filter; [b]$P<0.02$ against DX-free control on filter, combined with fibroblasts; [c]$P<0.001$ against fibroblast-free control without DX; [d]$P<0.001$ against fibroblast-free control with 0.25 μM DX; [e]$P<0.001$ against fibroblast-conditioned medium without DEX pretreatment; *DX was removed 6 h before conditioning commenced (see text).

A549 cells growing in direct contact with LF113 fibroblasts did not show any increased activity in either fibroblastic or steroid induction of PS relative to separated co-culture. The fibroblast plus steroid effect was, if anything smaller. Data are corrected for the presence of fibroblast protein (see Materials and methods), but the precise contribution of fibroblastic protein in direct co-culture is not known.

The activity of serum-free medium which had been conditioned by fibroblasts (FCM), with or without pretreatment with 0.25 μM DX, was determined on A549 cells grown in filter wells. After 48 h incubation, FCM from untreated fibroblast gave higher levels of PS production than unconditioned medium, and this was increased further by pretreatment of the fibroblasts with DX (Table II). These results confirmed that LF113 cells released a soluble factor(s) which could induce PS production in A549 cells, and that the production of this factor could be enhanced by DX.

HPLC analysis of glucocorticoid treated LF113 cells showed a rapid fall in the glucocorticoid per cell and was undetectable ($<5$ ng/$10^6$ cells) 2 h after steroid removal (data not shown).

This suggests that glucocorticoid carry-over into conditioned medium is negligible.

### *Partial purification of MOG-LF113 conditioned medium*

When ammonium sulphate precipitated fractions of FCM were assayed for stimulation of PS synthesis, the highest activity was observed in the 60% fraction (Figure 2a). Fractionation of FCM on a Biorad preparative isoelectric focusing gradient apparatus gave highest activity in fraction 12, equivalent to pH 8.8 (Figure 2b). Cation-exchange chromatography followed by precipitation with 60% ammonium sulphate were therefore chosen for steps in partial purification.

**Figure 2** Fractionation of conditioned medium. **a**, Ammonium sulphate precipitation. Conditioned medium was precipitated with increasing concentrations of $(NH_4)_2SO_4$, and filter well cultures assayed with precipitates reconstituted in PBSA. 'S' is the final supernatant after the final precipitation step. Each point is the mean of four replicates. **b**, Isoelectric focussing. Conditioned medium was fractionated on a Biorad Rotofor as described in the Methods, and the fractions assayed in filter well cultures of A549. Each point is the mean of three replicates. **c**, The pH of the gradient in **b**, is plotted in the bottom figure.

Dialysed conditioned medium from glucocorticoid-treated fibroblasts was applied to S-Sepharose as described in the Materials and methods and eluted with 1.0 M NaCl. The first eight fractions, shown to contain the bulk of the activity (data not shown) were pooled and the protein precipitated with 60% (w/v) $(NH_4)_2SO_4$. The resulting precipitate was designated FDF. A dose response curve for induction of PS with FDF is shown in Figure 3, showing that maximum activity had still not been reached by 1 $\mu g\,ml^{-1}$.

*Physico-chemical characterisation*

Isoelectric focusing (see above and Figure 2) indicated a pK of 8.8. Heating to 56°C for 30 min or 100°C for 5 min destroyed about 20% of activity (Table III). Acidification to pH 3.0 destroyed 90% of activity while increasing the pH to 9.0 had little effect. Digestion with 100 $\mu g\,ml^{-1}$ trypsin (Worthington, recrystallised) or 100 $\mu g\,ml^{-1}$ pronase (Sigma, *S. griseus*) removed 50% of the activity. This suggests that

**Figure 3** Induction of surfactant production by FDF. FDF, purified by S-Sepharose and ammonium sulphate precipitation as described in the Methods, was added to confluent filter well cultures of A549 and surfactant synthesis assayed after 3 days. Each point is the mean and standard error of three replicates.

**Table III** Sensitivity of FDF to proteases, acid, alkali and heat

| *Treatment* | *Concentration* | *Time* | *Temp °C* | *% of control* |
|---|---|---|---|---|
| Trypsin | 100 $\mu g\,ml^{-1}$ | 2 h | 37 | 50 |
| Trypsin + Soyabean inhib | 100 $\mu g\,ml^{-1}$ 100 $\mu g\,ml^{-1}$ | 2 h | 37 | 90 |
| Pronase | 100 $\mu g\,ml^{-1}$ | 2 h | 37 | 56 |
| Heat | | 30 min | 56 | 77 |
| Heat | | 5 min | 100 | 81 |
| Acetic acid | 1.0 M | 1 h | 4 | 50 |
| Tris HCl, pH 9.0 | 0.5 M | 1 h | 4 | 95 |

FDF is stable to heat and alkali but acid labile and protease sensitive (Table III).

*Activity of other growth factors*

Eight other growth factors were assayed for surfactant induction at concentrations known to be active in other systems (Table IV). Of these only PDGF, bFGF and IGF-1 showed significant activity, but much less than FDF. Although FDF was present at 1.0 $\mu g\,ml^{-1}$ the bulk of this, as indicated by preliminary SDS-PAGE analysis, is probably inactive, contaminating, high molecular weight proteins.

The apparent activity of insulin was not statistically significant, and subsequent assays with insulin at a range of concentrations from 0.1–10 $\mu g\,ml^{-1}$ have not shown any stimulation (data not shown).

*Phenotypic effects of FDF* in vitro

*Differentiation* The data in Figure 3 indicate that FDF induces surfactant synthesis in A549 cells. This has been taken as evidence of differentiation.

*Growth and survival* Monolayer cloning was performed on A549 cells which had been pre-treated with 0.1 ng ml–10 µg ml FDF for 3 days. FDF had no effect on the clonogenic potential of A549, with an average plating efficiency of 40%

**Table IV** Effects of known growth factors on pulmonary surfactant production in A549 cells

| *Growth factor* | *Mol. Wt. (kDa)* | *Concentration* | *% Stimulation* |
|---|---|---|---|
| Control | – | – | 0 |
| TGF-α | 5.6 | 0.1 μM | 25 |
| TGF-β | 25 | 1.0 ng ml⁻¹ | 4 |
| PDGF | 32 | 3.0 μ ml⁻¹ (70,000 μ mg⁻¹) | 211** |
| bFGF | 14–18 | 10.0 ng ml⁻¹ | 83* |
| IGF-1 | 7 | 10.0 ng ml⁻¹ | 75* |
| Insulin | 5.7 | 1.0 μg ml⁻¹ | 138 |
| EGF | 6 | 0.1 μM | 31 |
| Bombesin | 1.6 | 0.1 μM | 15 |
| FDF | ? | 1.0 μg ml⁻¹ | 1178*** |

*$P<0.02$; **$P<0.01$; ***$P<0.002$. Each observation represents the mean of three separate experiments. Statistical analysis was carried out using the Student's *t*-test for paired samples.

(approximately 200 colonies per petri dish) in both control and treated cultures. There was no difference in colony size between treated and controls (data not shown).

A549 was able to grow well in semi-solid agar with a plating efficiency of around 33%. Pre-treatment of A549 cells in regular monolayer culture with FDF, 1.0 μg ml$^{-1}$, caused a significant reduction in colony formation in agar by approximately 30%.

*Plasminogen activator (PA)* PA activity was measured in both A549 and another human lung adenocarcinoma, WIL. Treatment of both cell lines with FDF caused a significant reduction in PA activity (Table V).

### *Effect of FDF on growth of A549 cells as xenografts*

FDF significantly inhibited the growth of A549 as established xenografts (Figure 4) and histology revealed extensive structural reorganisation in the treated tumour (Figure 5), with stromal infiltration and signs of glandular-like or duct-like formation not evident in controls. Observation of the gross morphology of the tumours excised after treatment showed not only a significant difference in size but also a marked reduction in blood supply, judging by the whiter appearance of the tumour.

### Discussion

This study examined the role of fibroblasts, and a fibroblast-derived factor, on the differentiation of A549 type II pneumocyte tumour cells *in vitro*, and the effect of the factor on growth *in vivo*. PS synthesis and, particularly, the response to glucocorticoid, were greater when A549 cells were cultured on a filter well membrane rather than on a conventional plastic substrate, possibly due to the establishment of polarity and/or higher oxygen tension, being nearer to the gas-liquid interface. In epithelial cells, the expression of complete differentiation requires the development of polarity within the cell (Sattler *et al.*, 1978; Chambard *et al.*, 1982), although to what extent polarity is achievable in transformed cells is as yet unclear. It has been reported that for the correct expression of differentiated features in normal lung, it is often necessary to have the cells growing close to the air-liquid interface, since this mimics more closely the *in vivo* situation (Van Scott *et al.*, 1986), so elevating the culture closer to the surface of the medium may have contributed to increased PS synthesis.

The current data confirm that fibroblasts participate in the response of alveolar type II epithelial cells to hydrocortisone, and that the tumour cell line A549 retains this indirect response to steroid. A549 cells do respond to steroid directly, but to a much reduced extent. The addition of fibroblasts to the dish underlying the A549 cells in the filter increased the response of the A549 cells to DX nearly 4-fold over that of A549 alone. This effect was not increased by direct contact

**Table V** Effect of FDF on suspension cloning and plasminogen activator activity

| *Cell line* | *FDF μg ml⁻¹* | *Colony number (mean±s.e.)* | *PE %* | *Change in PE* | *PA Activity*[a] *(mean±s.e.)* | *Change in PA* |
|---|---|---|---|---|---|---|
| A549 | 0 | 333±26 | 33 | | 4.73±0.49 | |
| A549 | 1.0 | 221±16* | 22 | −33% | 1.53±0.13* | −68% |
| WIL | 0 | – | – | | 13.16±0.09 | |
| WIL | 1.0 | – | – | – | 3.83±0.21** | −71% |

*$P<0.02$; **$P<0.002$, unpaired student *t*-test; [a]Activity in ploug units mg$^{-1}$ total cell protein. Abbreviations: FDF = fibroblast derived factor; PE = plating efficiency; PA = plasminogen activator activity.

**Figure 4** Effect of FDF on growth of A549 as xenografts. Mice received injections of FDF three times weekly, i.p., and the tumour volumes were determined by double caliper measurements at the times indicated. The difference at 11d is significant at $P<0.01$ ($n = 5$).

between the A549 cells and the fibroblasts, indicating that the major effect was due to a paracrine factor as previously described for normal developing lung (Smith, 1979). This was confirmed by the activity of fibroblast-conditioned medium which stimulated production of surfactant in A549 cells, and the activity of which was also increased by glucocorticoid.

The activity of the fibroblasts in the absence of DX suggests that they have constitutive synthesis and release a PS stimulating factor(s) which may be increased by DX or distinct from the DX induced effect. Other growth factors (PDGF, bFGF, and IGF-1) were shown to have some PS inducing ability and may be released by fibroblasts without steroid stimulation.

Although fibroblast conditioned medium was shown to be active, a significant increase in this activity had been detected in medium conditioned by glucocorticoid-treated lung fibroblasts. A simple purification procedure was undertaken in an attempt to further enrich the activity of the conditioned medium, and reduce the risk of steroid carry-over. However, HPLC analysis indicated that steroid was undetectable ($<5$ ng/10$^6$ cells) by 2 h after steroid removal, and conditioning commenced at 6 h. As the cell concentration during conditioning was approximately $2.0 \times 10^5$ cells ml$^{-1}$, the maximum possible concentration of steroid in conditioned

**Figure 5** Histology of xenografts. Tumours were excised 1 month post-implantation, after treatment for 12 days out of the last 14 days with FDF, 50 ng g$^{-1}$, or PBS placebo, fixed and stained with H&E as in the methods. **a**, control (75 ×); **b**, FDF treated (75 ×); **c**, control (300 ×); **d**, FDF treated (300 ×).

medium would have been $<1$ ng ml$^{-1}$ ($2.5 \times 10^{-9}$ M), previously found to be too low for induction of surfactant synthesis. In view of this and subsequent purification steps, it is unlikely that sufficient steroid remained to account for the activity of the partially purified FDF.

These experiments indicate the presence of a diffusible factor or factors, here called FDF, capable of stimulating PS production in A549 alveolar carcinoma cells. The activity of FDF was found to be heat and alkali stable but sensitive to acid and protease. From the review of the activities of other growth factors, the likeliest candidate would be PDGF. However, PDGF is stable in acid and is therefore unlikely to be the sole source of activity. Since bFGF and IGF-1 both showed some activity, it is possible, particularly in view of the heterogeneity of the material, that the total activity is due to a synergistic interaction of two or more already established growth factors. TGF-β was inactive and, in a recent report (Torday & Kourembanas, 1990) has been shown to antagonise the activity of FPF, the PS-inducing factor previously reported by Smith (1979). Further purification and characterisation is clearly required before it can be established that this is a new and unique factor.

Initial results from Smith (1979) suggested that the activity described in normal developing lung was in a 5–7 Kd fraction and was acid stable. Material from the present purification was dialysed with a retention of approximately 10 Kd, and was found to be acid labile, and may therefore be distinct from Smith's FPF.

Proteolytic digestion with trypsin and pronase only reduced the activity by 50%. Since trypsin cleaves only on the carboxyl side of lysine and arginine residues, it may still have left active peptides, however, this is less likely with pronase, and it is therefore possible that digestion was incomplete, or some of the activity is not protein based.

The induction of surfactant synthesis in this system was shown to involve the formation of characteristic multilamellar bodies (data not shown) and is generally associated with terminal differentiation of type II pneumocytes. Increased clonogenicity in suspension (MacPherson & Montagnier, 1964) and elevated PA levels (Rifkind *et al.*, 1974) have generally been associated with the malignant phenotype, although the expression of PA is by no means unique to tumour cells (Duffy & O'Grady, 1984; Camiolo & Greco, 1986). The associated depression in suspension cloning and PA, together with the induction of PS synthesis, suggest that a general phenotypic change is occurring, with a coordinated shift away from malignancy towards differentiation. The possibility of such a relationship agrees with the findings of Frame *et al.* (1984), who showed that in a panel of early passage glioma cell lines, less well differentiated cell lines expressed high PA levels, while a decrease in PA expression was observed in cells induced to differentiate with glucocorticoid and glia maturation factor.

FDF reduced clonogenicity in soft agar by about 30% without any effects in monolayer, suggesting specific inhibition of anchorage independent growth. This is in agreement with the observations of Shirasuna *et al.* (1988), who noticed a marked reduction, not only in colony number, but also in colony size when a salivary adenocarcinoma cell line was treated with WI-38 conditioned medium.

As the factor represses tumour growth *in vivo*, this also substantiates the observation that induced differentiation represses malignancy. This may occur by increased sensitivity to density limitation of growth and the withdrawal of terminally differentiated cells from the proliferative pool. Furthermore, since the tumour seemed to be less well vascularised, induction of differentiation may have led to a reduction in angiogenesis factors produced by the tumours.

The inhibition of PA activity produced by the WIL cell line, derived from an adenocarcinoma, suggests that FDF activity may not be restricted to alveolar carcinoma, and preliminary data from treatment of other tumours *in vivo*, as yet with very small numbers of animals, suggests that FDF may have activity with other non-small cell lung cancers and small cell lung cancer, but not with ovarian carcinoma.

In summary these results provide good evidence that foetal lung stromal cells have important paracrine effects on malignant lung epithelium. This is due, at least in part, to a diffusible factor, which can be isolated, and the existence of which may point to a possible mode of antitumour therapy which should have minimal peripheral toxicity. Analogous activity has been observed in prostate where fibroblasts can influence the secretory capacity of prostatic carcinoma in a paracrine manner (Djaklew *et al.*, 1990) and the appearance of columnar cells in prostatic carcinoma can be induced by genital tract mesoderm (Hayashi *et al.*, 1990), suggesting that this type of paracrine control may be widespread and potentially applicable to other types of carcinomas.

We thank Drs Graham Cowling, Onchar Singh, Gierish Shahand and others of Glaxo Group Research for assistance in preparation of the conditioned medium, protein purification, and electron microscopy, and Mr Tom Hamilton for help with the animal experiments. This work was supported by grants from Glaxo Group Research (VS) and the Cancer Research Campaign (RIF).

### References

BOHNERT, A., HORNUNG, J., MACKENZIE, I.C. & FUSENIG, N.E. (1986). Epithelial-mesenchymal interactions control basement membrane production and differentiation in cultured and transplanted mouse keratinocytes. *Cell Tiss. Res.*, **244**, 413.

BOUKAMP, P., RUPNIAK, H.T.R. & FUSENIG, N.E. (1985). Environmental modulation of the expression of differentiation and malignancy in six human squamous cell carcinoma cell lines. *Cancer Res.*, **45**, 5582.

BRADFORD, M. (1976). A rapid and sensitive method for the quantitation of microgram quantities of protein using the principle of protein dye binding. *Anal. Biochem.*, **72**, 248.

CAMIOLO, S.M. & GRECO, W.R. (1986). Plasminogen activator content of human tumor and adjacent normal tissue measured with fibrin and non-fibrin assays. *Cancer Res.*, **46**, 1788.

CHAMBARD, M., GABRION, G., VERRIER, B. & MAUCHAMP, J. (1982). Thyroid cell polarisation and the expression of specialised functions. In *Mechanisms of Growth and Development*, Alan R. Liss: New York, pp. 403-411.

CHEN, T.R. (1977). *In situ* detection of mycoplasma contamination in cell cultures by fluorescent Hoescht 33258 stain. *Exp. Cell Res.*, **104**, 255.

CUNHA, G.R., FUJII, H., NEUBAUER, B.L., SHANNON, J.M., SAWYER, L. & REESE, B.A. (1983). Epithelial-mesenchymal interactions in prostatic development. I. Morphological observations of prostatic induction by urogenital sinus mesenchyme in epithelium of the adult rodent urinary bladder. *J. Cell Biol.*, **96**, 1662.

DJAKIEW, D., TARKINGTON, M.A. & LYNCH, J.H. (1990). Paracrine stimulation of a neoplastic prostatic epithelial cell line by prostatic stromal cell proteins. *Cancer Res.*, **50**, 1966.

DUFFY, M.A. & O'GRADY, P. (1984). Plasminogen activator and cancer. *Eur. J. Cancer Clin. Oncol.*, **20**, 577.

FERGUSSON, R.J., CARMICHAEL, J. & SMYTH, J.F. (1986). Human tumour xenografts growing in immunodeficient mice: a useful model for assessing chemotherapeutic agents in bronchial carcinoma. *Thorax*, **41**, 376.

FRAME, M.C., FRESHNEY, R.I., VAUGHAN, P.F.T., GRAHAM, D.I. & SHAW, R. (1984). Interrelationship between differentiation and malignancy-associated properties in glioma. *Br. J. Cancer*, **49**, 269.

GIARD, D.J., AARONSON, S.A., TODARO, G.J., ARNSTEIN, P., KERSEY, J.H., DOSIK, H. & PARKS, W.P. (1972). *In vitro* cultivation of human tumors: establishment of cell lines derived from a series of solid tumours. *J. Natl Cancer Inst.*, **51**, 1417.

HAYASHI, N., CUNHA, G.R. & WONG, Y.C. (1990). Influence of male genital tract mesenchyme on differentiation of Dunning prostatic adenocarcinoma. *Cancer Res.*, **50**, 4747.

GOERKE, J. (1974). Lung surfactant. *Biochim. Biophys. Acta*, **344**, 241.

IMANISHI, J., HOSHINO, S., MATSUOKA, H. & 5 others (1983). Tumour degeneration by human embryonic fibroblasts and its enhancement by interferon. *Cancer Res.*, **43**, 4323.

KEDINGER, M., SIMON-ASSMANN, P., ALEXANDRE, E. & HAFFEN, K. (1987). Importance of a fibroblast support for in vitro differentiation of intestinal endodermal cells and for their response to glucocorticoid. *Cell Differen.*, **20**, 171.

McLEAN, J.S., FRAME, M.C., FRESHNEY, R.I., VAUGHAN, P.F.T., MACKIE, A.E. & SINGER, I. (1986). Phenotypic modification of human glioma and non-small cell lung carcinoma by glucocorticoids and other agents. *Anticancer Res.*, **6**, 1101.

MACPHERSON, I. & MONTAGNIER, K. (1964). Agar suspension culture for the selective assay of cells transformed by polyoma virus. *Virology*, **23**, 291.

MASON, R.J., NELLENBOGEN, J. & CLEMENTS, J.A. (1976). Isolation of disaturated phosphatidylcholine with osmium tetroxide. *J. Lip. Res.*, **17**, 281.

NEUBAUER, B.L., CHUNG, L.W.K., McCORMICK, K.A., TAGUCHI, O., THOMPSON, T.C. & CUNHA, G.R. (1983). Epithelial-mesenchymal interactions in prostatic development. II. Biochemical observations of prostatic induction by urogenital sinus mesenchyme in epithelium of adult rodent urinary bladder. *J. Cell Biol.*, **96**, 1671.

PODESTA, A.H., MULLINS, J., PIERCE, G.B. & WELL, R.S. (1984). The neurula stage mouse embryo in control of neuroblastoma. *Proc. Natl Acad. Sci.*, **81**, 7608.

POST, M., FLOROS, J. & SMITH, B.T. (1984). Inhibition of lung maturation by monoclonal antibodies against fibroblast pneumocyte factor. *Nature*, **308**, 284.

RIFKIN, D.B., LOEB, J.N., MOORE, G. & REICH, E. (1974). Properties of plasminogen activators formed by neoplastic human cell cultures. *J. Exp. Med.*, **139**, 1317.

ROWLEY, D.R. & TINDALL, D.J. (1987). Responses of NBT-II bladder carcinoma cells to conditioned medium from normal fetal urinogenital sinus. *Cancer Res.*, **47**, 2955.

SATTLER, C.A., MICHALOPULOS, G., SATTLER, G.L. & PITOT, H.C. (1978). Ultrastructure of adult rat hepatocytes cultured on floating collagen membranes. *Cancer Res.*, **38**, 1539.

SMITH, B.T. (1977). Cell line A549: a model system for the study of alveolar type II cell function. *Am. Rev. Resp. Dis.*, **115**, 285.

SMITH, B.T. (1979). Lung maturation in the fetal rat: acceleration by injection of fibroblast pneumocyte factor. *Science*, **204**, 1094.

SMITH, B.T. & FLETCHER, W.A. (1979). Pulmonary epithelial-mesenchymal interactions: beyond organogenesis. *Hum. Pathol.*, **10**, 248.

SHIRASUNA, K., MORIOKA, S., WATANI, K. & 5 others (1988). Growth inhibition and differentiation of human salivary adenocarcinoma cells by medium conditioned with normal human fibroblasts. *Cancer Res.*, **48**, 2819.

TADERERA, J.V. (1967). Control of lung differentiation *in vitro*. *Dev. Biol.*, **16**, 489.

TORDAY, J.S. & KOUREMBANAS, S. (1990). Fetal rat lung fibroblasts produce a TGF$\beta$ homolog that blocks alveolar type II cell maturation. *Dev. Biol.*, **139**, 35.

VAN SCOTT, M.R., YANKASKAS, J.R. & BOUCHER, R.C. (1986). Culture of airway epithelial cells: research techniques. *Exp. Lung Res.*, **11**, 75.

WESSELLS, N.K. (1977). *Tissue Interactions and Development*. Menlo Park, CA: Benjamin Cummings.

WHUR, P., MAGUDIA, M., LOCKWOOD, B.J. & WILLIAMS, D.C. (1980). Plasminogen activator in cultured Lewis lung carcinoma cells measured by chromogenic substrate assay. *Br. J. Cancer*, **42**, 305.